# Mentoring & support practices for final year medical students during a pandemic – ‘The covid doctors’

**DOI:** 10.1186/s12909-023-04513-9

**Published:** 2023-07-26

**Authors:** Gurjot Brar, Sarah Harney, Oliver McGarr, John McFarland

**Affiliations:** University of Limerick, Limerick, Ireland

**Keywords:** Final year medical students, COVID-19, Pandemic, Transition

## Abstract

**Background:**

Transition from final-year medical student to newly graduated doctor is challenging with evidence of associated increased patient mortality and medical errors. Previous work suggests tackling preparedness alone does not ‘solve’ this transition. The current focus on mentoring and support provision during this period and is an under-researched area. The COVID-19 pandemic represents a unique disruptive critical incident in which to examine mentoring and support practices, exposing strengths and weaknesses. The perspectives of this cohort and their implications remains an under-researched area.

**Methods:**

Individual semi-structured interviews were conducted with nine graduate-entry final-year medical students. An inductive latent phenomenological approach explored individual experiences of mentoring and support practices during final-year and transition to professional practice.

**Results:**

Three major themes emerged: 1) Mentoring & Support; 2) Clinical Exposure; 3) Graduation & Transition. A journey metaphor was used to aid the description of participants’ lived experience of mentoring and support practices during their final year. Final year medical students (FYMs) felt under-supported and found practices inadequate. Reduced clinical exposure yielded unpreparedness and regression, potentially impacting future careers. Positive experiences were variable and unstructured. ‘The COVID Doctors’, subtheme provided rich insights into shared narratives and identities amongst participants.

**Conclusions:**

This study provides qualitative evidence for perceived inadequate mentoring and support provision for final year medical students at transition during a critical incident (the COVID-19 pandemic). Several themes using the metaphor of a journey explore the lived experience of this unique cohort determining their perceptions on the delivery of their medical education and their identity as ‘covid doctors’. There are several implications for this study in a post-pandemic era and for pandemic-preparedness, both rapidly growing areas of research in medical education. Recommendations include updating contingency plans, balancing clinical exposure with patient safety issues, and providing support to ‘bottom-up’ mentoring practices.

**Supplementary Information:**

The online version contains supplementary material available at 10.1186/s12909-023-04513-9.

## Background

Transition from final-year medical student to newly graduated doctor is a time of challenge and stress [1–3]. Studies have found evidence of increased patient mortality [4] and medical errors [5] associated with this transition. In addition to medical students feeling under-prepared [6], schools have been criticised for not suitably equipping students for medical practice [7].

Furthermore, a focus on preparedness alone does not ‘solve’ transition [8]. Current emphasis has developed towards mentoring and support provision, whilst reframing the transition as socio-culturally embedded ‘critically intensive learning periods’ (CILPs) [8]. Although a limited area of study, mentoring and support have been identified as central to reducing the negative impacts of transition [8]. Additionally, mentorship and support provision are key in facilitating legitimate peripheral participation [9], supporting zones of proximal development [10, 11].

The recent COVID-19 pandemic constituted a unique, destabilising, and protracted event in human history [12]. This event represented a large-scale critical incident defined as [13] *“…any sudden and unexpected incident…which causes trauma within a school community, and which overwhelms the normal coping mechanisms of that school.”* Such critical incidents represent timely opportunities to re-examine the integrity of a system, exposing any underlying strengths or weaknesses, particularly with respect to systems and communications.

Clinical experience at undergraduate level eases transition and equips students for their roles as doctors, [1–3]. Therefore, significant disruption in clinical placements due to the pandemic potentially renders this cohort more vulnerable at transition. The associated negative effects of transition on mental health and well-being have been documented both previously [14–16], and contemporaneously during the pandemic [17–19]. Additionally, the impact has been examined globally, [20–24] identifying numerous negative effects of the pandemic on final year medical students’ lives and career prospects. How universities responded during this critical incident has the potential to shape the future of medical careers. Preliminary research has identified approximately one-fifth of medical students believed the pandemic would affect their choice of specialty, specifically limitations in exploring specialities and obtaining recommendation letters for areas of interest [21].

Although previous research has demonstrated significant negative impacts of the pandemic on final-year medical students [17–24], a gap remains regarding a rich and detailed understanding of the lived experiences of this unique cohort. Examining mentoring and support practices during a critical incident may expose hidden strengths and weaknesses. This has the potential for expediting reform and enhancing focused supports for final year medical students during transition. Additional support in this crucial period has potential to reduce medical errors, burnout and negative effects on mental health whilst preserving professional identity formation and development. This study aims to provide qualitative data on the phenomenological experiences of a sample of final-year medical students during a critical incident (the COVID-19 pandemic) using an inductive latent approach.

## Methods

An inductive latent interpretative phenomenological approach was chosen for this study. Phenomenology concerns itself with the study of an individual’s perceived experience which has been inspired by philosophers such as Husserl who describe identifying the essence of a phenomenon without attending to preconceptions. Phenomenologists attempt to capture how individuals make meaning in their contexts and environments. Hermeneutics allows researchers to attempt to understand the participants’ meaning-making through their own accounts. This blended approach allows not only for the participants lived experience to be described, but also results in further insights and perspectives beyond those provided by the participant [25, 26]. As such, the complex and unprecedented circumstances for current final year medical students necessitated rich and detailed inquiry. An inductive approach links themes directly to the data and assumes researchers have some ideas of expected emergent themes (clinical exposure and transition to intern), providing depth and specificity to the data. Using this framework, themes that aggregate outside the researchers’ assumptions are also accommodated for [27]. A latent context adds detail to semantic data by deeper analysis, revealing hidden narratives, assumptions and discourses that provide richer interpretive layers via a constructionist paradigm. Using interpretive phenomenological analysis, insights can be drawn by researchers, not readily apparent to participants, allowing wider ranges of discussions to the fore [27].

### Context

Participants were final-year students in the 4-year graduate entry-medicine programme at the School of Medicine, University of Limerick during academic year 2020/2021. During the pandemic, several restrictions put in place by the government affected this cohort asymmetrically, most pertinently suspension of clinical placements. Internship follows graduation in Ireland, akin to the first year of the Foundation Programme (UK) and Postgraduate Year-1 (PGY-1) elsewhere. Final year assessment is continuous, culminating in high-stakes end-of-year examinations in each discipline. GB and JMcF came in proximal contact with the students during the six-week Psychiatry rotation and final examinations only. SH taught participants during years one and two only and OMcG had no contact with the participants.

### Ethical approval and process

University of Limerick Research Ethics & Governance Committee granted ethical approval (2020_12_09_EHS). GB conducted all semi-structured interviews, generating and anonymising transcripts prior to analysis. No risks were identified throughout the study using risk assessments conducted as part of the ethical approval process.

### Recruitment

One hundred forty-seven final-year medical students at University of Limerick received emails via gatekeepers to participate in semi-structured interviews. Age range 26–45 years, 64% from EU States and 59% female. Nine final year medical students (eight female) were recruited by voluntary participation through convenience sampling. All were contacted by GB once they had replied with written interest to the gatekeeper. All participants possessed primary degrees and had graduate-entry status. Participants’ primary degrees (ranging from Economics to Health-related sciences), ethnic backgrounds (Irish, European, and Non-European) and ages (26–45 years) were diverse. Interviews ranged between 41 min and 5 s and 56 min and 39 s and were conducted between 15^th^ February 2021 and 29^th^ March 2021. Written information was provided regarding the study and all participants provided informed consent prior to engaging in the interviews. Participants were reminded that they could withdraw consent at any time and participation was completely voluntary. While the overall response to the invitation was low, all participants provided insightful and relevant data as they were all pursuing their studies during the course of the pandemic. The overall demographic profile of this sample was reflective of the overall cohort of students that enrol on the program annually, with the exception of gender ratio (the class was 58% female and study participants were 88% female). Analysis of the text also indicates that a level of data saturation was achieved by the 6^th^ participant as the themes emerging were common across the different participant interviews.

### Data collection

The interview schedule, conceived by GB, SH and OMcG through several meetings, was piloted by GB and JMcF. GB conducted semi-structured interviews with each participant during the spring semester of 2021. All interviews were conducted and recorded via Microsoft Teams, transcribed verbatim and anonymised. The recordings of the interviews were then promptly destroyed. All participants provided respondent validation of final transcripts and themes.

### Data analysis

Data collection and analysis occurred simultaneously in iterative fashion. Each transcript was fully analysed, and first-pass coded before progressing to the next [28]. GB was immersed and highly familiar with the data, through collection and transcription. The coding process involved multiple researchers and produced emergent themes managed by the software ‘Dovetail App’. The first round of coding involved aggregating disparate data, later unifying into emergent themes. Superordinate themes began to emerge at this stage. SH, OMcG and JMcF analysed two transcripts and regular meetings were held to discuss divergence and convergence. All investigators met regularly to discuss identified themes. Themes were then grouped into strata providing final overarching themes. Data were further explored to delineate subthemes and reinforce superordinate themes. An audit trail and memo were kept throughout the duration of analysis. Assumptions of the researchers were identified and made explicit at the outset, initially recognised at the first meeting, and then revisited at points of extreme divergence/convergence. To avoid transferring assumptions, researchers regularly questioned and challenged each other to support reflexivity.

## Results

Three major themes emerged: Mentoring & Support; Clinical Exposure; Graduation & Transition.

The final year through to intern was represented as a journey to participants, requiring guides (mentors), infrastructure and waypoints (support) and training by rehearsal and tacit experiences (clinical exposure), reaching a conclusion where ‘the real challenge begins’ (transition to intern and beyond).

### Superordinate themes

#### Mentoring & support

All students identified a perceived lack of support and many discussed deficiencies in adequate mentoring. It appears the ‘journey’ proved difficult due to reduced infrastructure and waypoints, and a relative lack of contact with experienced guides.

##### Communication

Almost all students expected timely, tailored, and transparent communication with a human element, however, several were frustrated by a perceived disconnect:

*“I felt the human part was all taken out of the communication this year… sometimes those emails. You forget that there's a human behind it.” (Niamh)*^1^

*“I don't know what the policy was, I was so confused…the worst thing about being a medical student is feeling like a fool the whole time.”*

*“There's a complete disconnect this year, when there should be more of a connect in any other year” (Seamus)*

##### Contingency planning

Participants expected universities to have contingency plans in the event of critical incidents.

*“I feel like Covid was the main excuse this year for everything. It wasn't just a pandemic, it was an excuse. For not getting things done for us or for not supporting us or for lots of the different things” (Aisling)*

The possibility of contracting COVID-19, with subsequent absences, and impact on progression produced added tensions to the university-student dynamic:

*“It would kind of be your onus, so I feel like if that were to happen, that would be on you and the medical school naturally would try to separate itself from that.” (Caoimhe)*

##### Role models/mentors

There was evidence of strong guidance and mentoring in particular circumstances:

*“I had a lot of respect for him. He took regulations very seriously…made sure that his whole team wasn't going into rooms if not necessary…. gowned up the right way,…he was really nice and despite everything would involve me, ask me to present cases…actually listen …which as a student you don't get acknowledged by a lot of consultants”(Laura)*

However, in other instances, pandemic guidelines impeded rapport and familiarisation with informal and tacit learning experiences:

*“We weren't even able to go for coffee afterwards because everything was always closed. Yeah, so there's nowhere to even sit and get your mentoring!” (Aisling)*

##### Peer support

There was evidence of increased cohesion amongst the cohort, underpinned by the pandemic, which provided shared meaning, context and understanding of the individual lived experience:

*“…No one understands except for the group of people who actually are experiencing it…That's great for me, I have that support system. There are students who don't…and aren't doing too well…” (Caoimhe)*

#### Clinical exposure

Clinical exposure was heterogenous, inconsistent and varied among participants. Here training by rehearsal and tacit experience was particularly haphazard, relaying a disenfranchised outlook amongst participants on their journey.

##### Reduced clinical exposure

There were several examples of pandemic restrictions impeding clinical exposure for participants:

*“Anytime my team would go to ED to see patients. I was sitting on the chair outside for like 2 hours waiting for them to come back.” (Laura)*

*“I saw more C sections last year during my anaesthesia week than during my actual obstetrics rotation this year.” (Laura)*

Reduced exposure also impacted tacit learning:

*“It's all the skills, even the skills you take for granted that you need to learn, like communication…seeing how people talk to people…seeing how conversations are held, even to do with consenting...” (Aisling)*

Additionally, many participants pondered on potential changes in career trajectories:

*“I feel even more confused of what I want to go into because we didn't get that experience.” (Jenna)*

Some participants went as far to report feeling regressed:

*“One of the cliché sentences going around amongst us GEMS*^2^* is…we were nearly more proficient in 3*^*rd*^*-year than 4*^*th*^*-year…which says a lot” (Seamus)*

##### Variance

All participants described large variance between definition and enforcement of policy pertaining to safety protocols amidst the COVID-19 pandemic.

*“at the same time my housemate who was on medicine was allowed do all those things in the same hospital.” (Aisling)*

Participants also discovered high variance in teaching content, with almost all participants describing heterogeneous experiences in placements:

*“I thought the teaching in Paediatrics was good. I thought it was structured and…we got in to see children” (Seamus)*

*“Especially as I haven't seen children at all…it was a major catastrophe. All clinics are online. You wouldn't see any children.” (Eva)*

##### Extra clinical exposure

Some participants described success when using initiative to obtain extra clinical exposure and some positives were observed, such as increased organisation or ‘compensatory teaching’:

*“We weren't sure if we were allowed to shadow an intern, but I shadowed my intern because we weren't exactly told no and it's a really good experience.” (Aisling)*

*“Consultants would take you…they know that you haven't had enough exposure…show you more with the patient. It was more personalized…we would get attention from the consultants directly or more one-to-one teaching.” (Eva)*

#### Graduation and transition

All participants identified a lack of preparedness and formal transition period which led to the development of a shared narrative (‘The COVID Doctors’).

##### Preparedness

All participants expressed unpreparedness, but were divided in their belief regarding the significance of the impact of the pandemic:

*“I think everyone says they never feel prepared leaving, so I don't think I can blame that one on Covid.” (Niamh)*

*“No, I'm not sure whether it's related to COVID or not. I think it's just because medical school doesn't necessarily prepare you to work as an intern.” (Eva)*

Due to a lack of direct exposure, many participants described emergencies and procedural skills as specific areas of unpreparedness:

*“What if I'm that one person who gets the cardiac arrest bleep on day one? What am I gonna do like? anaphylaxis and things like that. I've never seen this, a lot of things in medicine I just haven't seen” (Caoimhe)*

##### Formal transition period

All participants acknowledged the transition period as arduous and proposed a formal transition period in response:

*“It’s always meant to be a difficult transition period…intern is a bit of a ‘baptism of fire’ anyway…It's a sink or swim kind of situation…I think it’s going to be one hell of a wakeup call.” (Niamh)*

*“you'd really hope that HSE*^3^* would realize maybe actually interns need a week of induction or two weeks of shadowing the person whose job they're taking over” (Niamh)*

*“maybe a two-week orientation, bring you in before like, I'd be happy to go in before for two weeks and not be paid just to catch-up on things, understand the system.” (Aisha)*

Several participants also channelled frustration at the ‘overnight’ change in status and responsibility:

*“So, for some reason a switch goes off when we qualify to finally step into those wards. It would be nice going into a continuing pandemic to see what a covid patient looks like…it's ridiculous that it's almost like they don't exist to students 'cause you can't see anything” (Caoimhe)*

##### ‘COVID Doctors’

Almost all participants referred to a shared identity of doctors who spent their final years disrupted by the COVID-19 pandemic. Referring to this identity, participants attempted to anticipate expectations placed on them:

*“I'm going on the fact that I hope everybody else in there is cognizant of the fact that we are the ‘covid year’. You know we're those ‘covid doctors’! It’s going to be stressful. It's going to be horrific, but hopefully it won't be too bad.” (Niamh)*

Several participants described a ‘catching-up’ period, believing they were ‘behind’ even before starting and that a potential stigma of their shared identity would remain:

*“You know, like babies when they are born a little bit pre-term, they start doing things a little bit like 2-3 weeks later. I think that's what will happen to us.” (Eva)*

*“Well, it's gonna take time to catch-up…I'd always be worried there will be a stigma of being like ‘oh you’re a 2021 doctor’ or you’re a ‘COVID doctor’. Will that last? 'cause I feel like medicine is that kind of place that it could last.” (Aisling)*

## Discussion

Conceptualising the pandemic as a critical incident allowed for re-examination of mentoring and support provision, exposing inherent strengths and weaknesses. Collectively, the findings suggested that participants felt underequipped to deal with the transition from graduation to internship.

Specifically, FYM’s demonstrated a sense of being under-supported and identified a perceived lack of adequate mentoring and support, with a compensatory increased dependency on peer support. Additionally, participants felt underprepared and regressed due to reduced exposure, believing this would impact their future career trajectories. ‘Compensatory teaching’ and sporadic positive mentoring occurred, but this appeared highly variable and unstructured. Communication and contingency planning were highlighted as particularly deficient areas of support by participants.

Interestingly, a key subtheme, ‘The COVID Doctors’, provided a rich insight into the narrative and development of a shared identity amongst participants. FYMs further described potential difficult transition periods and stigma as a result.

The findings of this study align with previous research, particularly themes surrounding transition and preparedness [8, 29–32], and point to a disconnect between FYM expectation and institutional provision. There was a clear perception of a dysfunctional compensatory system that was unable to deliver adequate mentoring and support. It was apparent that participants expected explicit communication and contingency plans in the face of uncertainty. A possible cultural shift was noted, in line with the observation that the identity of current students is increasingly consistent with that of a “consumer”, [33].

Nevertheless, positive role models/mentors and increase in peer support was observed. FYMs reported ‘compensatory-teaching’ and ‘bottom-up’ mentoring and support, although usually sporadic and reliant on student initiative. Notably, these positive experiences were perceived as resulting from individual efforts rather than a cohesive effort from academic staff within the university. Variance in policy and teaching content was reported by many participants, alluding to commencement/suspension of clinical placements at various times during the year in relation to changes to pandemic restrictions.

The Covid-19 pandemic appears to have had a largely disruptive effect on FYMs. Both as a critical incident, revealing fragilities in current mentoring and support provision; and as a pandemic, directly impacting FYMs and their futures. FYMs were divided in their belief that the pandemic impacted their career trajectories but were unified in identifying potential stigma and issues at transition.

With a notable sense of agency and alignment through legitimate peripheral participation [9], FYMs envisioned a role for graded exposure, criticising the abrupt overnight change associated with status and responsibility [29]. The public expect newly qualified doctors to be ‘oven ready and self-basting’ [34], which may now extend to deal with patients with COVID-19 sequelae. However, the current study indicates that due to policy adherence and reduced exposure, students have not had opportunities to learn from patients symptomatic of COVID-19. Correspondingly, a pre-pandemic study described students were rarely able to practice managing acutely ill patients due to patient-safety concerns [1]. Participants observed the somewhat paradoxical expectation to be treating such patients shortly after graduation.

FYMs expected formal transition periods, placing such large emphasis on its importance by deciding to take on this experience without compensation. It was evident that, at an early stage in their trajectory, participants acknowledged that the expectations and responsibilities of the doctor-role comes before learning. This observation lends support to the theory of transition beginning earlier than graduation [35], representing a prolonged developmental process [30]. Our findings support the evidence that “sub-internship” may improve self-assessed preparedness in this regard [31, 36, 37]. Furthermore, FYMs anticipated the need to cope with pressures and uncertainty regardless of its challenges, adding to their burden of stress [1], “bracing for an experience to be endured, rather than enjoyed” [29]. Concerningly, there is evidence that the ‘transition shock’ which accompanies abrupt changes in role and responsibilities [29, 38, 39], produced by disparities between expectations and role-transitions [8, 30, 40], can lead to the development of dysfunctional strategies [29, 41].

Previous research suggests the presence of inadequate mentoring and support during the transition period, not only at provision [30] but also in terms of access [29, 30, 40]. The observations in the current study are in-line with previous work suggesting that students may be reluctant to ask for support, for fear of appearing vulnerable and ‘imperfect doctors’ [40]. Effective mentoring and support practices represent complex solutions to the complex problem of transition, particularly in the face of critical incidents. Effective supervision and role-modelling are essential factors in professional identity formation and the development of clinical competence but require balancing with societal needs [42, 43]. As performance of newly graduated doctors is contingent to a large extent by organisational practices and cultures, situated and relational mentoring and support practices can be considered vital [8] to engender sociocultural learning processes and progressive participation in practice [9]. Thus, stakeholders reframing the transition period as critically intensive learning periods (CILPs) may allow students to better engage with the idiosyncrasies of the workplace environment [8]. In alerting stakeholders of the challenges faced by this cohort during transition, there is potential for expectations to be tempered and access to and provision of adequate mentoring and support enhanced. Indeed, as one-third of trainees report being affected by burnout during the COVID-19 pandemic [44], significant cultural change may be required to create environments that foster effective mentoring and support practices.

### ‘The COVID doctors’

The ‘COVID doctors’ subtheme provided a rich insight into the participants’ construction of a shared narrative in relation to social identity theory [45].

Participants anticipated transition to be challenging, choosing to present themselves in a vulnerable way, leaning on the narrative to manage expectations of colleagues, supervisors and possibly themselves. This impression management [46] may serve a further purpose in managing expectations of future career trajectories. Furthermore, FYMs appeared to require validation of their unique status, as a rationale for perceived deficiencies in clinical knowledge/skills when undergoing the transition from a learning to a performance orientation [30]. The pre-term infant metaphor may extend beyond the ‘catching-up period’, having implications for professional development and identity formation. It can be assumed this narrative is not static and may serve as a performance management tool for this cohort to navigate this transition. For instance, FYMs may present a resilient facet of this shared identity, describing how challenges were overcome in their final year as students. These challenges may retrospectively be made to fit narratives of strength, creativity and overcoming adversity [47]. The findings suggest the utility or stigma of the narrative is likely to be context dependent. Nonetheless, a disrupted final-year remains a potential risk factor for increased medical errors and mortality associated with transition [41].

This study highlights how a critical incident affected FYMs undergoing transition to intern and exposed underlying strengths and weaknesses in their medical education. There are several implications for this study in a post-pandemic era and for pandemic-preparedness, both rapidly growing areas of research in medical education [48–51]. Recommendations include updating outdated contingency plans [48, 51], balancing clinical exposure with patient safety issues [49], and providing support to ‘bottom-up’ mentoring practices [48].

### Strengths & limitations

This study provides rich, detailed and contextually novel perspectives of FYMs in transition during a critical incident. Methodologically, rigour was applied in reflexivity and researcher triangulation. Our intentions are the rich description allows readers to interpret this study in relation to their practice, indubitably also affected by the COVID-19 pandemic.

The sample may not represent views of all, but data saturation with nine participants is considered acceptable [52]. Interviews were conducted by a member of faculty known to students. Although all precautions were taken in relation to power dynamics, complete omission remains impossible. Thus, it remains possible interviewees were not fully honest during their accounts, potentially leaving layers to these perspectives uncovered.

## Conclusions

This study provides qualitative evidence for perceived inadequate mentoring and support provision for final year medical students at transition during a critical incident (the COVID-19 pandemic). Several themes using the metaphor of a journey explore the lived experience of this unique cohort determining their perceptions on the delivery of their medical education and their identity as ‘covid doctors’. There are several implications for this study in a post-pandemic era and for pandemic-preparedness, both rapidly growing areas of research in medical education. Recommendations include updating contingency plans, balancing clinical exposure with patient safety issues, and providing support to ‘bottom-up’ mentoring practices.

## Supplementary Information

**Additional file 1:** **Appendix 1.** Superordinate Themes. **Appendix 2.** Intermediate Themes (size indicates number of themes). **Appendix 3.** Initial to Superordinate Themes.

## Data Availability

The datasets generated and analysed during the current study are not publicly available due to being interview generated and anonymised transcripts but are available from the corresponding author on reasonable request.

## Abbreviations

FYM: Final Year Medical Student
GEMS: Graduate Entry Medical School/Student
CILP: Critically Intensive Learning Period
COVID-19: Coronavirus Disease 2019

## Footnotes

1 To facilitate immersion whilst maintaining anonymity, pseudonyms are used from here on to present results.

2 GEMS – Graduate Entry Medical School – University of Limerick. (Former name). Now officially known as the School of Medicine. Students are colloquially referred to and self-refer as ‘GEMS’.

3 HSE – Health Service Executive – Irish Public Healthcare System.
