# Supplementary material for: Mentoring & support practices for final year medical students during a pandemic – ‘The covid doctors’
Source: BMC Med Educ. 2023 Jul 26;23:534. doi: 10.1186/s12909-023-04513-9 (PMC10373250; doi:10.1186/s12909-023-04513-9)
Supplement: Supplementary file 1 — Additional file 1: Appendix 1. Superordinate Themes. Appendix 2. Intermediate Themes (size indicates number of themes). Appendix 3. Initial to Superordinate Themes. [file 12909_2023_4513_MOESM1_ESM.docx]

| **Appendix 1 – Superordinate Themes** | | | | |
| --- | --- | --- | --- | --- |
| **Participant** | **Mentoring & Support** | **Clinical Exposure** | **Graduation & Transition** | **Total** |
| 1 | 19 | 36 | 22 | 77 |
| 2 | 31 | 25 | 31 | 87 |
| 3 | 18 | 12 | 5 | 35 |
| 4 | 13 | 15 | 8 | 36 |
| 5 | 17 | 12 | 9 | 38 |
| 6 | 13 | 8 | 8 | 29 |
| 7 | 30 | 13 | 8 | 51 |
| 8 | 19 | 9 | 12 | 40 |
| 9 | 30 | 9 | 12 | 51 |
| **Total** | **190** | **139** | **115** | **444** |

Table 1. Overall number of codes for each participant and major themes

Several themes emerged from the data but the three above will be the focus of this paper. Auxiliary themes relating to mental health, isolation and frustration have been reported elsewhere and thus were not the focus of this study.

**Appendix 2 – Intermediate Themes (size indicates number of themes)**


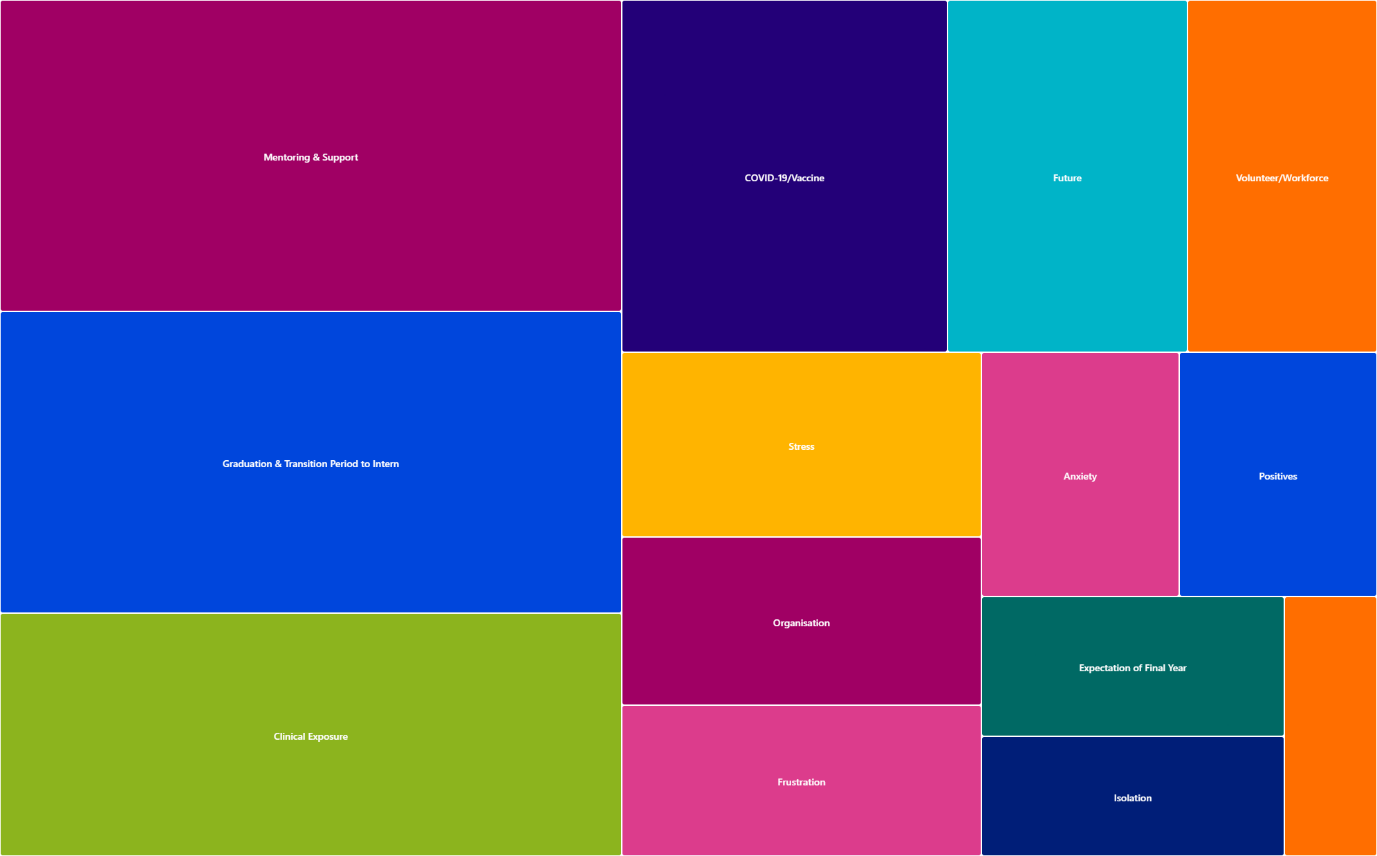

Appendix 3 – Initial to Superordinate Themes
